# Mechanical tumor microenvironment and transduction: cytoskeleton mediates cancer cell invasion and metastasis

**DOI:** 10.7150/ijbs.44943

**Published:** 2020-04-27

**Authors:** Xingchen Li, Jianliu Wang

**Affiliations:** 1 Department of Obstetrics and Gynecology, Peking University People's Hospital, Beijing, 100044, China; 2 Beijing Key Laboratory of Female Pelvic Floor Disorders Diseases, Beijing, 100044, China

**Keywords:** metastasis, cytoskeleton, mechanical force, TRP channel, mechanotransduction

## Abstract

Metastasis is a complicated, multistep process that is responsible for over 90% of cancer-related death. Metastatic disease or the movement of cancer cells from one site to another requires dramatic remodeling of the cytoskeleton. The regulation of cancer cell migration is determined not only by biochemical factors in the microenvironment but also by the biomechanical contextual information provided by the extracellular matrix (ECM). The responses of the cytoskeleton to chemical signals are well characterized and understood. However, the mechanisms of response to mechanical signals in the form of externally applied force and forces generated by the ECM are still poorly understood. Furthermore, understanding the way cellular mechanosensors interact with the physical properties of the microenvironment and transmit the signals to activate the cytoskeletal movements may help identify an effective strategy for the treatment of cancer. Here, we will discuss the role of tumor microenvironment during cancer metastasis and how physical forces remodel the cytoskeleton through mechanosensing and transduction.

## Introduction

Metastasis is the process by which cancers migrate to a distant organ and develop into a metastatic lesion^1^. However, cancer cells are unable to accomplish this process alone. The tumor microenvironment (TME) also is known to play an essential role in tumor metastasis 2. Reciprocal biochemical and biophysical interactions among tumor cells, stromal cells and the extracellular matrix (ECM) result in a unique TME that determines disease outcome. The cellular component of the TME contributes to tumor growth by providing nutrients, assisting in the infiltration of immune cells, and regulating the production and remodeling of the ECM 3. The TME consists of surrounding blood vessels, the extracellular matrix, secreted soluble factors, and other stromal cells 4, 5.

Mechanical forces within the TME play a pivotal role in driving physiological and pathological processes of cancers 6. These forces have been identified as critical components of the TME and coordinate their behaviors during various biological processes, including cell division, survival, differentiation and migration 7, 8. In solid tumor, mechanical force is caused by an elevation in the structural constitutions, particularly in the amount of cancer cells, stromal cells, and EMC components. With the increasing number of the cancer and noncancerous cells, the pressure inside the tumor rises and the signals of mechanical forces transfer to cancer cells, leading to mechanotransduction and cancer progression 9. There are many types of stresses from TME could be loaded to cancer cells including substrate rigidity, fluid shear stress, hydrostatic pressure, and tensile and compressive forces 10.

Mechanosensing describes a cell's ability to sense mechanical cues from its microenvironment, including not only force, stress and strain, but also substrate stiffness, topography and adhesiveness. This ability is critical for cells to react to the surrounding mechanical cues and adapt to the varying environment 11. Various mechanical signals are detected by and transmitted to the cells through activation of superficial mechanosensors such as integrins, G protein-coupled receptors (GPCR), transient receptor potential (TRP) ion channels, Piezo channels and YAP/TAZ 12-16. The TME provides changing mechanical cues to the mechanoreceptors of cancer cells, which convey the signals to their internal machinery and affect the cellular behaviors. This communication process is called mechanotransduction and taking place in a continuous feedback cycle 17. Mechanotransduction translates mechanical stimuli into biochemical signals, changing gene expression or regulating the cytoskeleton and membrane traffic, to ultimately alter cellular functions 18.

In response to mechanosensors, the cytoskeleton, an intracellular architecture composed of microtubules, microfilaments, and intermediate filaments that together determine the mechanical properties of cells, undergoes dramatic changes 19. Cells are intricately connected to the external environment through their cytoskeleton, which receives external signals that guide complex behaviors such as lamellipodia formation, invasion and migration 20. Whereas the contribution of chemical signals in the TME has long been understood, mechanical signals have only recently been widely recognized to be pervasive and powerful 21. The cytoskeletal structure plays an integral role in transducing external mechanical signals to internal responses 22.

Physical forces mediate the cytoskeleton through mechanosensors by activating various pathways, such as GTP-binding protein RhoA 23, the Hippo pathway, the focal adhesion kinases (FAK), JAK/STAT, and PI3K-AKT pathways et al. Knowing the pathological mechanical force and signaling pathways is critical for selecting therapeutic strategies for metastatic cancers.

In this review, we will discuss recent progress towards an integrated understanding of the mechanical TME and its physical influence on cancers. Furthermore, we especially focus on how these mechanical signals transmitted by mechanosensors influence metastasis through cytoskeletal structures.

## Influence of TME and mechanical properties of TME on tumor progression

Solid tumor is consisted of a complicated mixture of cancer cells and noncancerous cells. Overall, these noncancerous cells together with factors including the extracellular matrix, cytokines, growth factors, and hormones, make up the tumor microenvironment 24. The major constitutions of TME include vascular, CAFs, immune cells, TAMs, tumor-associated endothelial cells, and ECM 25. TME has an influence on the entire process of tumors from initiation to metastasis. What's more, tumor cells in turn influence the biochemical and biophysical properties of the TME to make TME conductive to the growth of tumor 26. Variations in physical aspects, such as matrix stiffness, geometry, gradients of soluble factors, and electromagnetic fields are also features of the tumor microenvironment 27.

Within the last decade of cancer research, it has been shown that mechanical stimuli in the TME affect cells as profoundly as chemical signals do. Multiple substrates in TME produce or transmit mechanical signals to cancer cells, thus leading to the cancer cells acquiring features that are primarily focused on invasion and metastasis 28. In this review, we concentrate on three components that play essential roles in the TME: ECM, TGF-β and CAFs.

### ECM regulates cytoskeleton and metastasis through substrate stiffness in TME

ECM provides biochemical signals and mechanical support, which can both sustain cellular constituents. Fundamentally, the ECM is composed of proteoglycans (PGs), glycoproteins (GAGs) and fibrous proteins such as collagen, elastin, fibronectin (FN) and laminin, which are controlled by ECM and provide cancer cells with mechanical support 29. Rather than serving simply as a physical support, the ECM is a physiologically active component of living tissue, responsible for cell-cell communication, cell proliferation and metastasis 30.

It has previously been proposed that tumors are stiffer than their surrounding normal tissue, and tumor stiffness is mainly determined by the amount of ECM, particularly collagen and hyaluronan 31. ECM stiffness may cause intracellular contractions and a subsequent increase in the stiffness of an actin cytoskeleton that favors cancer migration 32. Increasing ECM stiffness also induces malignant phenotypes, characterized by Rho-dependent cytoskeletal tension that leads to enhanced cell-ECM adhesions, disruption of cell-cell junctions and increased growth 33. ECM stiffening can also enhance the connection between the ECM and the cytoskeleton through local adhesion proteins, and increase cytoskeletal tension by Rho/ROCK signaling activation 34. The ECM also regulates cytoskeletal tension in hepatocellular carcinomas 35.

In addition, stiffening of the ECM is accompanied by an incremental increase in collagen deposition and a progressive linearization and thickening of interstitial collagen, which induces tumor aggression and causes immune cells to infiltrate tumor cells 36. As the collagen receptor and the transmembrane connector between cellular cytoskeleton and ECM, integrin allows the transmission of forces, which the cells generate with their actomyosin onto the ECM 37, 38.

Matrix stiffening can also activate TGF-β signaling, which mediates epithelial-mesenchymal transition (EMT), leading to the acquisition of a more aggressive phenotype that promotes cancer metastasis 39. EMT contributes pathologically to cancer progression through signaling pathways and among these, the actin cytoskeleton has played a predominant role 40. Cells that undergo EMT reorganize their cortical actin cytoskeleton into one that enables dynamic cell elongation and directional motility 41. Therefore, dysregulation of ECM composition and stiffness contributes to changes in cytoskeletal structure and enables metastasis 42.

### TGF-β regulation of the cytoskeleton in Epithelial-Mesenchymal Transition

Furthermore, the TME also sequesters and locally releases growth factors including epidermal growth factor (EGF), fibroblast growth factor (FGF), insulin-like growth factor-1 (IGF-1), vascular endothelial growth factor (VEGF), and other signaling molecules, such as WNTs and transforming growth factor-β (TGF-β) 43. ECM components release these factors through ECM cleavage, and these factors also regulate ECM architecture and influence cell behaviors 44. Mechanical stimuli can result in the local release or activation of the above-mentioned factors stored in the ECM 45. Among all these factors, TGF-β is also stored in the matrix as part of a large latent complex and can be activated by cell contractile force. Matrix straining and stiffening lower the threshold for TGF-β activation by increasing the mechanical resistance to cell pulling 46.

TGF-β regulates the actin cytoskeleton in cancer cells, mainly by promoting EMT, an event of reorganization of cytoskeleton architecture and dissolution of the epithelial cell-cell junctions 41. TGF-β-induced EMT is characterized by dramatic changes in cytoskeletal structure mediated in part by changes in the expression and organization of cytoskeleton proteins, including intermediate filaments, microtubules and microfilaments 47. In carcinoma, increased expression and activation of TGF-β promotes the epithelial plasticity response, which leads to cancer cell invasion and dissemination 48. Binding of TGF-β family proteins in cancer cells activated SMAD signaling pathway 49, which controls disruption and rearrangement of the actin-cytoskeleton 50. In response to TGF-β, SMAD signaling not only activates the expression of EMT transcription factors such as SNAIL, MRTF and ZEB, but also increases their activity 51-53. TGF-β also induces the activation of SMAD-independent pathways like Rho GTPases, p38MAPK and ERK1/2 54, and drives actin reorganization and formation of lamellipodia and filopodia 55-57. For example, one study showed that TGF-β induced activation of the RhoA-LIMK2-cofilin-1 pathway to modulate the actin cytoskeleton by increasing actin polymerization in colorectal cancer cells 58. In non-small cell lung cancer, TGF-β induced EMT and migration by activating cytoskeleton microtubules and the functions can be attenuated by RCCD1 depletion 59. It has been shown that TGF-β also causes changes in the cytoskeletons of gastrointestinal and prostate cancer cells 60, 61. Tight junctions connect adjacent cells and associate with the intracellular actin cytoskeleton. TGF-β dissolves tight junctions and down-regulates the potential proteins including claudins, occludins and ZO1 62.

In TGFβ-induced cytoskeletal rearrangement, miRNA participates in the process. For example, miR-155, which is expressed in response to TGF-β, targets the mRNA encoding RhoA, resulting in the dissolution of tight junctions 63. TGF-β also induces the expression of miR-24, which targets neuroepithelial cell-transforming 1A (NET1A), a RHO-GEF that activates RhoA, therefore promoting EMT through the disruption of adhering and tight junctions 64.

### CAF produces growth factors, chemokines and extracellular matrix to facilitate metastasis

By dynamically interacting with tumor cells, stromal cells participate in all stages of tumor initiation, progression, metastasis, recurrence and drug response, and consequently, affect the fate of patients. During the processes of tumor evolution and metastasis initiation, stromal cells in TME also experience some changes and play roles in both the suppression and promotion of metastasis. However, the overall function of stromal cells is beneficial for cancer cell survival and movement 65. Stromal cells in the TME include endothelial cells, smooth muscle cells, pericytes, fibroblast cells and immune cells. Fibroblasts that are found in the TME are called Cancer-Associated Fibroblasts (CAFs) and can be identified by expression of certain markers, including α-smooth muscle actin (α-SMA), fibroblast activation protein (FAP) and fibroblast-specific protein-1 (FSP1) 66. A large percentage of tissue fibroblasts are transformed to CAFs that contain high levels of α-SMA. Therefore, it is proposed that TGF-β activates fibroblasts to become CAFs, which in turn produce more ECM fibers leading to desmoplasia 67.

During metastasis, CAFs are recruited by cancer cells and lead the way ahead of cancer cells to promote proliferation and migration by alignment of the actin cytoskeleton 68, 69. They generate force to reorganize the matrix through a Rho-mediated myosin function, allowing them to clear the path for the cancer cells. Induction of the YAP transcription factor is required for the ability of CAFs to remodel EMT to support tumorigenesis. YAP induction in turn regulates multiple factors that modulate the cytoskeleton and matrix stiffness 70. Expression of multiple cytoskeletal regulators is activated by YAP, which enables the fibroblasts to stiffen the surrounding matrix and promote cancer cell invasion 28. FXR activation inhibits the tumor stimulatory activities of CAFs by governing cytoskeleton organization, stress fiber formation and contractility 71.

CAFs become activated by several tumor-derived growth factors such as TGF-β, which increases stiffness of CAFs by reorganizing their cytoskeletons to increase their elongation, cell spreading, lamellipodia formation and spheroid invasion 72. TGF-β also enhances CAF formation, which is regulated by the rate of microtubule polymerization, depending on β-tubulin composition 73, 74.

CAFs are also the main contributors to ECM stiffness. CAFs interact with almost all cells within the TME and regulate cancer cell cytoskeletons indirectly through mediating EMC stiffness 75. Hypoxia and TGF-β are the key inducers for CAFs in regulating TME stiffness and they strongly influence tumor and stromal cell properties such as proliferation and motility 32, 76.

CAFs respond differently to diverse levels of substrate stiffness, mainly by secreting the α-SMA and thus causing cytoskeleton remodeling and tumor invasion 77. Mechanical stimuli also activate CAFs through MRTF-SRF and YAP-TEAD pathways interacting indirectly to control cytoskeletal dynamics 78. In another study, Cdc42EP3 also responds to mechanical stimulation and plays a role in CAFs through tight regulation by Cdc42, which is a key regulator of cytoskeletal organization through its effects on actin assembly, actomyosin contractility and microtubules 79.

**Figure 1:**
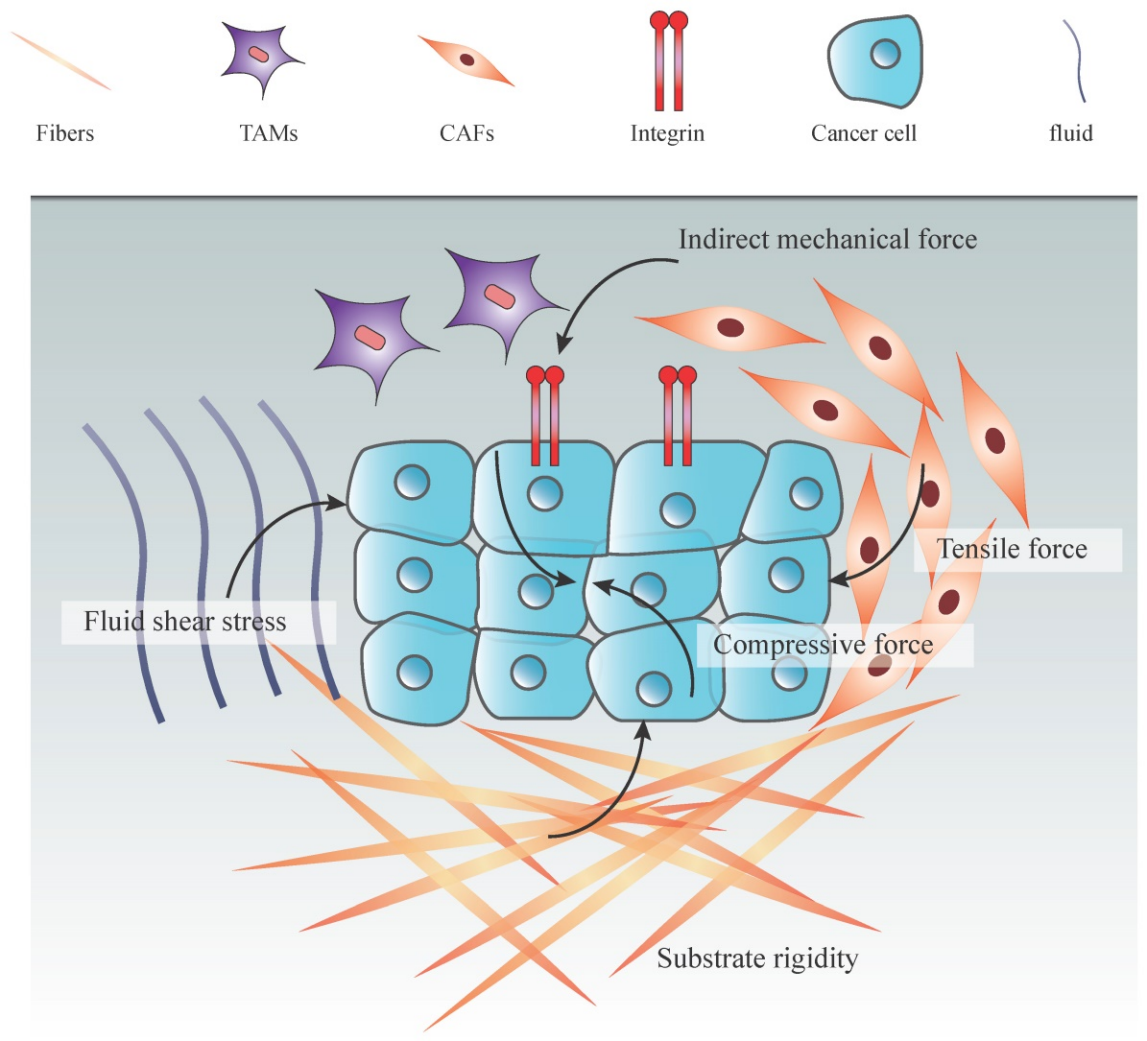
Types of mechanical forces in tumor microenvironment. An increasing number of cancer cells increas the solid stress among tumor cells, including tensile and compressive forces. Together with the ECM stiffening and proliferation of stromal cells, these forces promote the interstitial pressure. Moreover, fluid from leakage of blood vessels and secretion of stromal cells increase both fluid pressure and hydrostatic pressure. Indirect mechanical forces transduced by CAFs and TAMs to the mechanosensors (integrin) also play an essential role in the mechanical tumor microenvironment.

CAFs are known to possess the ability to reorganize the stromal cells by secreting ECM and enzymes that covalently cross-link collagen fibers, and by pulling the collagen network 80. What's more, the contribution of secretions from tumor-associated macrophage (TAM)-like cells to the stimulation of mechanical property changes in TME is also an important factor for stiffness of the ECM in cancer cells 81 (**Figure 1**).

## How cells sense mechanical signals and mediate the cytoskeleton

As we stated above, biomechanical elements, biochemical elements and stromal cells work together to control cancer cell fate during progression and are crucial for the maintenance of TME homeostasis. Loss of mechanical homeostasis in the TME accompanies tumorigenesis and also contributes to invasion and metastasis 82. Nevertheless, the biomechanical nature of TME is influenced by both biochemical cues and stromal cells indirectly. For example, the secretion of biochemical factors such as TGF-β and MMPs activates alterations in the biomechanical properties of the TME and remodeling of the ECM 83. Stromal cells can then regulate matrix alignment by releasing increasing amounts of proteases and auxiliary growth factors that trigger mechanical changes to the ECM 84, and CAFs are able to remodel the tumor matrix within the TME and provide the nutrients and chemicals to promote cancer cell invasion 85 and migration 86.

Progression of a tumor is characterized by increasing ECM stiffness. With stiffening of the ECM, the external force and plasticity of cancer cells increase 87. Meanwhile, the ECM is a source of biochemical and biomechanical signals that promote tumor progression, and it is in turn strongly influenced by the cancer in a reciprocal relationship that is driven by the cytoskeletons of cancer cells 34. Cells are equipped with several different mechanisms to sense the physical properties of the microenvironment and the mechanical forces arising within it 88. Mechanical forces in the TME can mediate the cytoskeleton and promote cancer cell migration in two ways: by directly transducing mechanical force via the cytoskeleton, or indirectly by mechanosensors 89. These mechanosensors translate mechanical forces into biochemical signals that trigger changes in the structure of cancer cell cytoskeletons 90. Then, at the cellular level, cancer cells actively respond to externally applied forces and then couple to intracellular signaling pathways and effectors 91. For instance, integrin-mediated adhesion of cells to matrix stimulates the activity of Rho GTPases and actin remodeling to regulate cell contractility and modify cellular behaviors such as survival and migration 92.

### Mechanical force remodels the cytoskeleton directly

The cytoskeleton senses and transduces mechanical stress directly and the extracellular force mainly produced by the ECM conversely regulates cytoskeletal formation and structure 93, 94. The principal components of the cytoskeleton include the actin filaments, microtubules and intermediate filaments 95. Mechanical forces impact all three components of the cytoskeleton.

Previous reports have indicated that when a mechanical force is applied to cancer cells, the actin filaments act as a mechanosensor that senses the mechanical forces 96, 97. They play a crucial role both in generating contractile forces by combining with the motor protein myosin II and by polymerization, which pushes the plasma membrane forward 98. The pitch length of helical actin filaments was increased by the tensile force, thus reducing the affinity of cofilin and increasing the affinity of myosin II to the actin filaments 99. Cancer cells interact with extracellular tension through this mechanism, which also helps them regulate cell proliferation and gene expression 100. The extent of actin filament alignment and the direction of the applied force relative to this alignment are key determinants of mechanotransduction efficiency 101.

Microtubules (MTs) are highly dynamic structures involved in cellular growth, vesicular formation and especially mitosis. MTs are critical for mediating mechanical force-directed spindle organization and chromosome alignment in mitosis 102. Tensional force at the surface of MTs is crucial for the mitotic spindle assembly checkpoint and the regulation of orientation and positioning 103. Cell migration requires the involvement of MTs in the formation of pseudopodia responding to mechanical cues from TME, and IncRNA may participate in that progress 104. Although these studies reveal the role of MTs in mechanoresponse, there is no direct evidence that MTs act independently as mechanosensors.

Compared with the mentioned actin filaments and MTs, intermediate filaments (IFs) are relatively stable filaments as the third component of the cytoskeleton, and are also flexible with the ability to be stretched 2.6-fold by tensile force 105. Due to the stable and resilient nature of IFs, they serve as an essential component in sensing the strength and direction of mechanical forces endured by cancer cells 89. It remains unclear whether IFs act independently as mechanosensors, but they are believed to be involved in mechanical response. Further studies are required to confirm the mechanisms of IF mechanical response.

### Mechanical force-induced cytoskeleton modification indirectly by cellular mechanosensors

**Figure 2:**
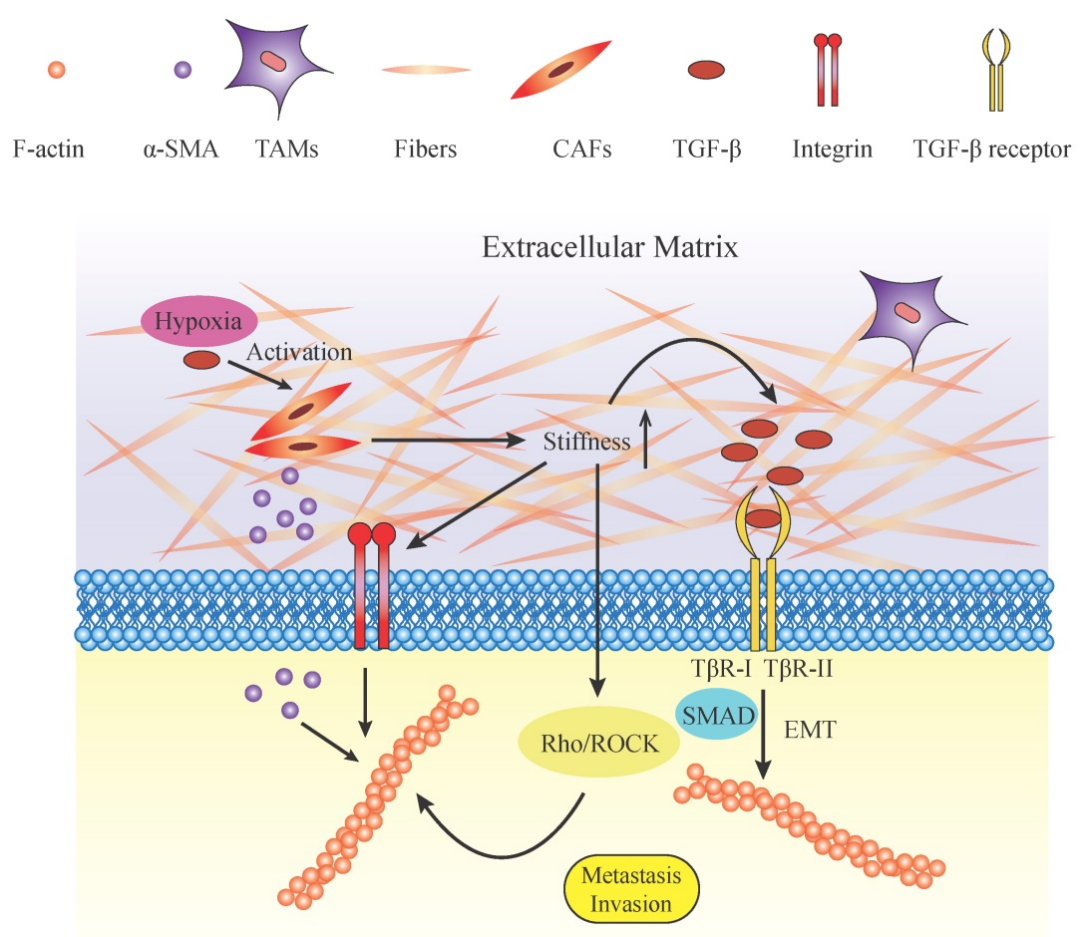
Components and roles of mechanical microenvironment in regulating cytoskeleton. Potential roles of biomechanical and biochemical factors in tumor microenvironment (TME). The TME embeds tumor cells, cancer-associated fibroblasts (CAFs), tumor-associated macrophages (TAMs), and their derivatives. The mechanical environment is mainly composed of extracellular matrix and its components such as proteoglycans (PGs), glycoproteins (GAGs) and collagenous fibers. The increased ECM deposition elevates its stiffness and rigidity. With the increasing rigidity of the ECM, mechanical force applied on tumor cells also increases. Mechanical force activates tumor cell progression directly by exerting pressure on the cell membrane or indirectly by putting force on integrins. The Rho/ROCK pathway is subsequently activated and contributes to the reorganization of F-actin. In addition, TGF-β and other growth factors are stored within the ECM and released in a tension-dependent manner. TGF-β combines with its ligands TβR-I/TβR-II. TGF-β regulates the actin cytoskeleton by promoting EMT, which is characterized by dramatic changes in cytoskeletal structure through the SMAD pathway. TGFβ-SMAD signaling activates the expression of EMT transcription factors, and SMAD complexes cooperate with these transcription factors to increase their transcriptional activities. In another way, TGF-β and hypoxia activate the CAFs which release α-SMA. α-SMA is transduced to the intracellular plasma and accelerates the formation and organization of the cytoskeleton. These biomechanical and biochemical parameters act together and lead to the reorganization of the cytoskeleton and the formation of lamellipodia, which act as the motile force and stimulate tumor cell invasion and metastasis.

Mechanical force must be transduced to an intracellular signaling pathway in order to influence cell behavior. Cancer cells contain several mechanosensing components that jointly connect the ECM with the cytoskeleton, thus transducing mechanical signals into biochemical cascades 106. Cellular mechanosensing is based on force-induced conformational changes in mechanosensitive proteins subjected to molecular forces. These changes result in opening of transmembrane channels or altered affinities to binding partners, thereby activating signaling pathways (**Figure 2**).

#### Integrins

Cell-ECM interactions in both normal and pathological conditions are principally mediated via integrins. The ability of cells to sense ECM stiffness can be attributed to the integrins 107. Considering that the communication between cancer cells and ECM takes place mainly through the ECM, the involvement of integrins in the mechanotransduction is significant 108. Integrin-mediated adhesions interact with the ECM and sense its rigidity, which in turn regulates cellular behaviors such as motility and migration 109. One critical mechanosensory response that underlies migration is the strengthening of ligand-integrin-cytoskeleton linkages under mechanical forces 110. Integrins regulate cytoskeletal organization and activate intracellular signaling pathways, conveying both mechanical and chemical signaling.

The binding of integrins with specific components of the ECM initiates outside-in signaling that eventually triggers the regulation of the cytoskeleton, while the mechanical forces generated by the cytoskeleton can be transmitted to the integrin-ECM interaction causing cancer metastasis 111. Integrin-mediated mechanotransduction also modulates gene expression via the nucleoskeleton 112. Recent study revealed that physical deformation of the membrane, either by mechanical force or curvature, can induce integrin activation 113.

#### YAP/TAZ

Recently, the transcriptional regulators YAP and TAZ were found to be the key mediators of cell growth and differentiation activated by matrix rigidity 114. These proteins can localize to the nucleus where they interact with transcription factors and promote expression of a number of genes involved in cell growth and differentiation 115. Corruption of cell-environment interplay leads to aberrant YAP/TAZ activation, which is instrumental for multiple cell behaviors including cancer proliferation, metastasis and stemness essential for tissue regeneration 116.

YAP/TAZ localized in the cell membrane is directly regulated by ECM stiffness, cell shape and cytoskeleton tension, which are required for YAP/TAZ nuclear localization 114. For example, YAP/TAZ is mechanically activated when they are cultured on the stiff ECM 117. Several different mechanisms and molecules have been implicated to explain mechanotransduction, such as membrane dynamics, nuclear mechanics, Hippo signaling and the Rho pathway 118. Integrins also have been reported to be the central transducers of mechanical cues from the ECM 119. Tumorigenesis requires increased force transmission between oncogene-expressing cells and their surrounding extracellular matrix, and these regulations rely on YAP/TAZ mechanotransduction 120.

Given the critical connection between cell mechanics and YAP/TAZ activity, it is not surprising that YAP/TAZmay also function to reinforce the cytoskeleton and the mechanical properties of ECM responses to mechanical stress 121. Expression of several regulators and components of the actomyosin cytoskeleton, including myosin IIB, myosin regulatory light chain 2 and filamin A, also is enhanced by YAP 122, 123. Furthermore, YAP/TAZ activity is tightly coupled to actin cytoskeleton architecture and enhances the membrane-cytoskeleton integrity resulting in the viability of cancer cells during metastasis 124.

The reciprocal regulation between YAP/TAZ and cytoskeletons allows us to understand how cells translate and influence mechanical stimuli during the promotion of survival, migration and metastasis.

#### Transient Receptor Potential (TRP) ion channels

Transient receptor potential (TRP) cation channels represent a large and diverse family of ion channels that is sensitive to multiple environmental factors such as temperature, light and mechanical force both at organismal and cellular levels 125-127. Discovery of these channels has greatly increased our understanding of mechanotransduction pathways in a variety of fundamental cell processes, and they are recognized as potential biomarkers for many types of cancer 14, 128, 129. The majority of TRP channels are non-selective and mainly calcium-permeable. Dysregulation of intracellular calcium gating or expression of the TRP family causes deregulation of downstream effectors, thus promoting proliferation and metastasis of cancer cells.

Several TRP channels are gated from opening by the application of certain mechanical forces, and thus function as stretch-activated channels 130. A mechanical stimulus could exert its influence on a TRP channel directly or alter the membrane tension that in turn indirectly opens the channel. For instance, mechanical tension elevates cAMP and activate PKA, thereby enhancing the mechanosensitive activation of some TRP channels associated with cell migration 131. TRPM7 functions as a part of mechanosensory complex driving metastasis, formation and invasiveness of breast cancer cells, involving the kinase domain of the channel and phosphorylation of MAPK 132.

Mechanical force transmitted through adhesion sites can be translated into biochemical signals by TRP channels, which may serve to localize signal transduction pathways as well as intracellular cytoskeletal dynamics 133. TRP channels are engaged in a reciprocal interplay with the cytoskeleton and control the TME for cytoskeletal dynamics 134. However, they can also be regulated by the cytoskeleton. For instance, TRPM7 is involved in regulating myosin II-based cellular tension and modifying focal adhesion (FA) through F-actin and paxillin 135. The interaction between TRPC1 and the calcium sensor STIM1 depends on an intact actomyosin cytoskeleton 136. The bidirectional regulation between TRP channels and the cytoskeleton occurs in multiple signaling pathways. TRPM7 controls actomyosin dynamics by phosphorylation of cytoskeleton components in a kinase-dependent manner 137. TRPV4 modifies the cytoskeletons in breast cancer cells, allowing them to migrate. This occurs via Ca^2+^-dependent activation of AKT and down-regulation of E-cadherin cell cortex proteins 138. Other TRP subfamilies also play important roles in cancers through various mechanisms 139. These findings provide new insight into the role of TRP channels in tumor progression and cytoskeletal structure.

#### Piezo1/2

Piezo channels are key force sensors, which sense the mechanical signals and transduce mechanical stimuli into intracellular signals typically by increasing cytosolic Ca^2+^ concentration 15, 140. Piezo channels have two main variants, Piezo1 and Piezo2. Piezo1 is shown to be a cation-selective channel that does not require ancillary proteins for its activity, and senses changes in the rigidity of the environment 141. Piezo2 is a critical regulator in tumor angiogenesis and vascular permeability 142. Piezo channels are regulated by cellular signaling pathways, typically by calcium influx 143. Piezo1 triggers a variety of intracellular processes, mainly related to cytoskeleton modulation. The actomyosin cytoskeleton exerts complex effects on Piezo1 activity and it in turn modulates cytoskeletal dynamics. Emerging data in the field support the idea for such a feedback loop 21. For Piezo2, it generates Ca^2+^ influx that triggers downstream activation of the RhoA-mDia pathway, which is necessary for regulation of the actin cytoskeleton 144. In conclusion, Piezo channels are closely associated with cytoskeletons and regulate their organization through calcium influx or interaction.

## Pathways regulating metastasis through the cytoskeleton

The cytoskeleton has mechanical functions that involve supporting the structural integrity of the cell (such as cellular shape, adhesion and motility), and non-mechanical functions that may include the regulation of cellular architecture, cell growth, and stress pathways. Cytoskeleton reorganization is induced by the activation of multiple signaling pathways to modulate diverse physiological and pathological processes including angiogenesis, proliferation and migration. Many signaling pathways have been reported to have an impact on actin cytoskeleton regulation through metastasis. The molecules in the pathways are major drivers of many of the steps required for metastatic success. In this section, we will focus on some key regulators in the signaling pathways controlling the cytoskeletal organization processes important for metastasis.

### RhoA/ROCK

One of the main cellular signaling pathways involved in regulating actin and cytoskeletons is the Rho family GTPase signaling pathway. This pathway has been identified as a fundamental contributor controlling several biochemical pathways underlying migration, such as cytoskeletal dynamics, directional sensing, cell-cell junction assembly/disassembly and integrin-matrix adhesion 20. To date, approximately 20 Rho GTPases have been reported in humans (of which Rho, Rac, and Cdc42 are the best studied), which orchestrate the remodeling of actin-containing cytoskeletal structures and regulate the cell contractile machinery to control many cellular processes 145.

Ras homolog gene family member A (RhoA) plays a key role in the regulation of actin polymerization, basement membrane disassembly and cortical contractility 146. Several proteins, including Rho-associated protein kinase (ROCK), formins, and other scaffolding molecules, have been identified as downstream targets of RhoA 147. ROCK, the major target of RhoA, directly phosphorylates Lim kinase (LIMK), an actin cytoskeleton regulator 148. LIMK also acts as an important downstream effector of Rho directly 149. Cofilin is a key regulator of actin severing, nucleation and capping within protrusive machinery. The phosphorylation of cofilin at Ser3 blocks the actin binding interface, preventing its actin binding function and promoting F-actin stability and elongation 150.

Ras-related C3 botulinum toxin substrate 1 (Rac1) reorganizes the actin cytoskeleton to promote formation of large membrane protrusions, called lamellipodia, which drive motility in many cell types 151. Cell division control protein 42 homolog (Cdc42) promotes the formation of actin-rich microspikes to sense extracellular chemotactic gradients and initiates directed cell movement 152. Cdc42 signaling also can generate actomyosin contraction through p21 protein (Cdc42/Rac)-activated kinase 2 (PAK2) and Cdc42-binding kinase (MRCK) kinases, which are related to myotonic dystrophy kinases 153. Shear stress and Cdc42 activation are also sufficient to promote filopodia formation by adjusting cytoskeleton in cancer 154. Rho family GTPase play a crucial role in a range of human diseases and is now considered as a potential target for the treatment of several malignancies including gastric cancer 155, breast cancer 156 and prostate cancer 157.

Rho-ROCK signaling is a key regulator of actomyosin contractility and regulates cell shape, cytoskeletal arrangement and thereby cellular functions such as cell proliferation, differentiation, motility and adhesion. ROCK isoforms differentially modulate cancer cell motility by mechanosensing the substrate stiffness 158. Rho-ROCK signaling has been shown to promote cancer cell growth, migration and invasion 148. Pre-clinical evidence supports a role for Rho-ROCK signaling in enhancing the malignancy of cancers. Pharmacological inhibition of Rho-ROCK using either Fasudil or Y-27632 decreased the invasion, stress fiber organization and migration in pancreatic adenocarcinoma cells and breast cancer cells in vitro 159, suggesting a cell-autonomous role for Rho-ROCK signaling in tumor progression.

### G protein-coupled receptors (GPCRs)

G protein-coupled receptors (GPCRs) are a family of proteins able to promote changes in cell shape. To date, GPCRs transduce signals into rapid changes on the actin-cytoskeleton via the activation of small GTP-binding proteins of the Rho GTPases family, such as Rho, Rac and Cdc42. GPCRs also modulate cAMP levels in order to exert diverse effects on cytoskeleton remodeling 160. As the effector of cAMP, PKA induces stabilization of the microtubule cytoskeleton, resulting in the inhibition of RhoA activity, myosin light chain (MLC) phosphorylation, and actomyosin contractility 161. Activated myosin connects the actin filaments to form stress fibers that generate actomyosin force to facilitate cell movement.

Actin polymerization in response to GPCR activation may imply the recruitment of proteins capable of modulating the activation of RhoA-mediated pathways, which lead to the formation of actin stress fibers 162. Chemokine signals participate in the recruitment of cells during metastasis, promoting spreading of different types of tumors. Chemokine receptors coupled to GPCR proteins induce cell motility and actin reorganization, acting through activation of Rho, PI3K and MAPK signaling 163. GPCRs can also activate the Hippo mechanical pathway by pressure-controlled YAP regulation 164.

As a steroid-acting GPCR, G-protein estrogen receptor (GPER) has important transcription-dependent outcomes in the regulation of cell growth and programmed cell death secondary to GPER-regulated second-messenger pathways 165. GPER is expressed ubiquitously and has diverse biological effects, including regulation of proliferation and migration 166. Non-genomic transcriptional effects induced by estrogen regulate F-actin cytoskeleton assembly and breast cancer cell metastasis through GPER acting on the Rho/ROCK-LIMK-cofilin pathway 167.

### PI3K-mTOR-RhoA/Rac

Phosphatidylinositol 3-kinase (PI3K) plays a central role in a complex, multi-armed signaling network that orchestrates cell responses including cell proliferation, migration and glucose metabolism 168. PI3K is presumed to activate most of its downstream targets via the Akt protein, which phosphorylates diversified downstream substrates including the mammalian target of rapamycin (mTOR), a master regulator of protein translation 168.

mTOR signaling is activated in conditions of migration deregulation in many cancer types including breast, ovarian, renal and glioblastoma 169. mTOR is a downstream effector of the PI3K/AKT pathway and forms two distinct multiprotein complexes: mTORC1 and mTORC2. mTORC2 functions to regulate spatial aspects of yeast cell growth, by controlling the actin cytoskeleton 170. It is activated by cancer hallmarks, phosphorylates PKC-α, PI3K/AKT and regulates the activity of Rho GTPase, which is related to the regulation of the actin cytoskeleton. mTORC2 signals to the actin cytoskeleton by activating a RhoA GTPase switch. Upon activation, RhoA interacts and activates PKC1, which in turn signals to the actin cytoskeleton via the MAPK pathway 171. mTORC2 also signals to the actin cytoskeleton, and although the direct targets of mTORC2 are unknown, this signaling may involve PKCα and the small GTPases Rho and Rac 172. PI3K/AKT can also inhibit the mechanical and mechanosensing properties of tumor cells 173.

Hence, the mTOR pathway is a central coordinator of fundamental biological events, playing a key role in cell growth and regulation of the actin cytoskeleton and cell survival 174.

### The Hippo pathway

The Hippo signaling pathway regulates diverse physiological processes, and genetic deletion or aberrant expression of some Hippo pathway genes leads to enhanced cell proliferation, tumorigenesis and cancer metastasis 175. There are many upstream signals that regulate the Hippo pathway, among which rearrangement of the cytoskeleton exerts the strongest effects on the pathway by the action of cell-cell and cell-matrix junction components and by the mechanical properties of the ECM 176-178. The actin cytoskeleton is believed to play a critical role in relaying mechanical forces to Hippo signaling, but the exact biochemical mechanisms by which the actin cytoskeleton impacts the core kinase cascade remain poorly understood.

The Hippo kinase cascade converges on its nuclear effector Yki/YAP/TAZ to regulate gene expression programs. Phosphorylation of Yki/YAP/TAZ by Hippo signaling inactivates these transcriptional co-activators by excluding them from the nucleus, and additionally for YAP/TAZ, by promoting their degradation 179. When Hippo signaling is low, Yki/YAP/TAZ enters the nucleus to drive gene expression. Other identified regulators of the Hippo pathway include mechanical cues, ligands of GPCRs, cell polarity, energy status, and hormonal signals 180.

### Ca^2+^ acts as the secondary messenger

Calcium is one of the most important elements for human beings and usually acts as a secondary messenger. Proper control of Ca^2+^ signaling through the actin cytoskeleton is mandatory and critical for cancer cell metastasis. At the cellular level, increases in Ca^2+^ trigger a wide variety of physiological and pathological processes. Thus, it is not surprising that aberrant Ca^2+^ signaling can induce malignancy for a broad spectrum of diseases.

Local Ca^2+^ pulses are generated from two sources, the internal Ca^2+^ storage and the external Ca^2+^ influx. Most internal Ca^2+^ signals originate from the endoplasmic reticulum (ER) through inositol triphosphate (IP_3_) receptors and extracellular calcium influx is induced by calcium channels of the TRP family, Piezo1/2, and Cav family, for example. Some of the cellular calcium signaling is triggered by mechanical force 181. Increased Ca^2+^ concentration in the cytoplasm is regulated locally and globally for effective cytoskeletal remodeling, cell migration and cancer metastasis. Ca^2+^ pulses and spikes occurring at particular locations and times activate numerous downstream structural and signaling targets 182.

Ca^2+^ signaling regulates the Rho GTPases 183, which are mandatory for the formation of actin bundles in lamellipodia, focal adhesion complexes, and filopodia, which are the major components for cell migration. Although the present data reveals no evidence of direct binding between Ca^2+^ and Rho GTPases, it is reasonable to expect their mutual interactions considering their perfect coordination during cell migration 184. It has been shown that constitutively active Rac1 fully counteracted the effects of SOC influx inhibition in migrating breast cancer cells 185, indicating the regulatory role of Ca^2+^ on Rho GTPases. Knockdown of the calcium channel reduces calcium influx, thus decreasing RhoA activity 144. There are many other Ca^2+^-related pathways that regulate cytoskeletal proteins in cancer, such as ERKs 186 and lipid signaling 187.

As we previously described, the oscillation of local Ca^2+^ pulses activate the Rho GTPases in the front of migrating cells and synchronizes with the retraction phases of lamellipodia 188. STIM1-ORAI1 also plays a key role in the control of Ca^2+^ entry at the leading edge of migrating cells 189, where this Ca^2+^ mobilization enhances the reorganization of the cortical cytoskeleton required for the formation of filopodia and lamellipodia 190.

**Figure 3:**
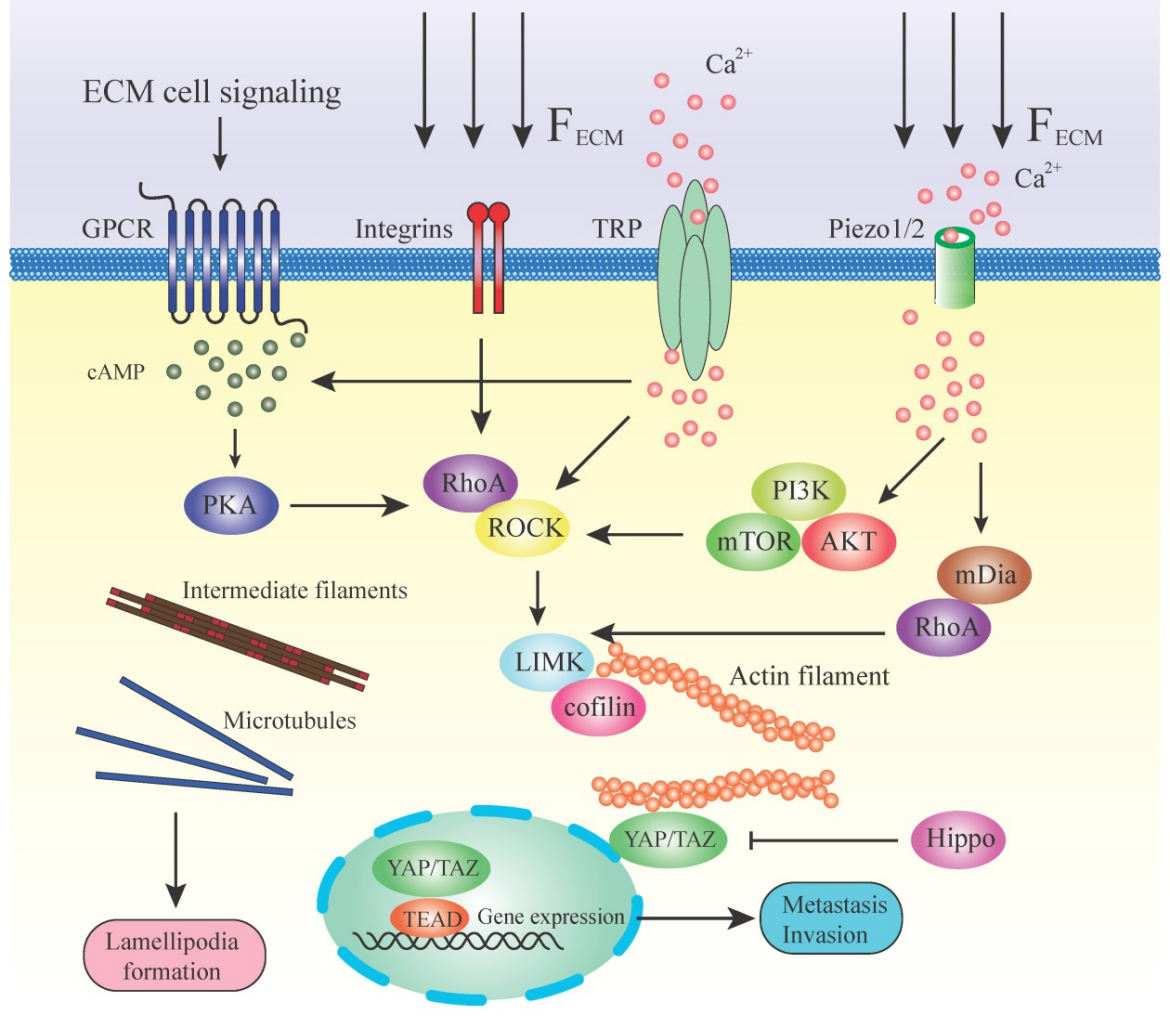
Mechanotransduction and pathways regulating cytoskeleton during metastasis. This illustration summarizes some of the mechanisms by which a stiff ECM or extracellular signaling alters cell fate through mechanotransducers. A stiffened matrix prompts cell tension and generates mechanical force, activating different mechanosensors. RhoA/ROCK is located in the center position among all these molecules in the pathways. RhoA is facilitated by numerous effector proteins. GPCR, via activation of cAMP and PKA, enhances the expression of RhoA. Integrins activate the RhoA directly by transducing the mechanical ECM force. TRP channels and Piezo 1/2 can sense the ECM force and lead to the release of Ca^2+^. Ca^2+^ influx stimulates RhoA or via PI3K/AKT/mTOR and mDia. Downstream of RhoA/ROCK lies the key regulators of cytoskeleton LIMK/cofilin. ROCK activates LIMK, which subsequently inhibits cofilin via phosphorylation. Cofilin facilitates actin filament severing and depolymerization; therefore, its inhibition results in elevated polymerized actin stability. LIMK also directly enhances actin polymerization and myosin contractility. This, in turn, initiates a paracrine signaling mechanism that causes increased production of ECM components in the tumor microenvironment. High expression of actin contractility and inhibition of the Hippo pathway activates YAP/TAZ function. YAP/TAZ accumulates in the nucleus and regulates gene transcription together with DNA‑binding transcription factors such as TEAD. ECM directly transduces the mechanical force to intermediate filaments and microtubules and assists in the formation of lamellipodia.

In addition, the F-actin severing protein cofilin also depends on the cytosolic Ca^2+^ for its proper activity. Moreover, myosin, as one of the major actin regulators, is totally dependent on Ca^2+^ for proper activity 191. Therefore, though not a direct regulator, Ca^2+^ modulates actin dynamics through multiple signaling pathways and structural molecules (**Figure 3**).

## Future perspectives

In this review, we reported on the critical role of the mechanical TME in cancer cell migration and the actin cytoskeleton. We focused on essential mechanisms by which mechanical signals produced by ECM and stromal cells in the TME promote cancer metastasis through the regulation of cytoskeletal dynamics and biomechanical and biochemical transduction occurring during this regulation. Under normal conditions, cell motility and cytoskeletal structure are tightly controlled in order to maintain cell physiological activity and tissue homeostasis. If either the mechanical microenvironment or the expression of mechanotransduction molecules is disturbed, the balance of the mechanical microenvironment and cell will have to be reorganized to restore equilibrium.

Often, structure, mechanotransduction, and cellular behavior are tightly linked. The current challenge that remains is to distinguish the multiple target markers in the process of the TME-cellular function relationship. In particular, there is still a lack of understanding of how the stromal components generate the physical force and transduce to modify the cytoskeletal reorganization and cellular functions such as cancer metastasis. What's more, another great challenge is lacking of methods to gauge the specific mechanical pressure during the whole progression of tumor. Understanding these processes may provide us with new clues in addressing the question of how cells manage to sense their physical environment and how we could intervene during the metastasis.

Understanding the basic mechanisms of mechanical properties in TME contributes to the progress of tumor treatment and this will bring hope to thousands of patients with cancer. Studying the mechanisms that underlie these processes and identifying key molecular targets will also lead us to new therapeutic strategies and further exploration of new therapeutic approaches based on the mechanical signals generated by tumors. It will also provide us with innovative treatment and potential curable strategy from another new point of view.
